# Carbapenem-resistant Gram-negative bacteria exhibiting clinically undetected cefiderocol heteroresistance leads to treatment failure in a murine model of infection

**DOI:** 10.3389/fmicb.2025.1496514

**Published:** 2025-05-09

**Authors:** Hongwei Liu, Peng Zhou, Peng Ma, Yaqin Liu, Yingfeng Zhang, Qiwei Li, Lingqing Xu, Wenchang Yuan, Weiguo Yin, Linhai Li, Yang Lu

**Affiliations:** ^1^The Affiliated Qingyuan Hospital (Qingyuan People's Hospital), Guangzhou Medical University, Qingyuan, China; ^2^Department of Public Health Laboratory Sciences, School of Public Health, Hengyang Medical School, University of South China, Hengyang, China; ^3^Department of Laboratory Medicine, The Second Affiliated Hospital of Guangzhou University of Chinese Medicine, Guangzhou, China; ^4^KingMed School of Laboratory Medicine, Guangzhou Medical University, Guangzhou, China

**Keywords:** heteroresistance, cefiderocol, gene mutation, failure treatment, carbapenem resistance

## Abstract

**Introduction:**

Carbapenem-resistant (CR) Gram-negative pathogens are classified by the WHO as critical threats due to limited therapeutic options. Cefiderocol (CFD), a novel siderophore cephalosporin, shows promise but remains unapproved in China. This study investigated the prevalence, clinical impact, and genetic mechanisms of cefiderocol heteroresistance (CFD-HR) in CR and ESBL-producing clinical isolates from China, where CFD remains unapproved.

**Methods:**

A total of 407 CR and ESBL-producing isolates were analyzed. CFD-HR was identified by population analysis profiles (PAPs). Clinical relevance was assessed through disk diffusion susceptibility testing, time-kill assays, and a murine peritonitis model. Genetic mechanisms and stability were elucidated by whole-genome sequencing (WGS) and fitness cost assays.

**Results:**

CFD-HR prevalence was 17.4% (16/92) in carbapenem-resistant *A. baumannii* (CRAB), 27.9% (24/86) in carbapenem-resistant *P. aeruginosa* (CRPA), 23.8% (10/42) in carbapenem-resistant *E. coli* (CRE), and ≤10% (1/10 in ESBL-producing *P. aeruginosa* and 8/177 in ESBL-producing *E. coli*). Although 72.9% (43/59) of HR isolates were classified as CFD-susceptible by disk diffusion, time-kill assays showed that 66.7% (4/6) of HR strains required ≥8 mg/L CFD (vs. 4 mg/L for non-HR) to prevent regrowth. *In vivo*, CFD achieved 100% (3/3) survival in non-HR infections but only 16.7% (4/6) in HR-infected mice. WGS identified transient genetic alterations in HR subpopulations, including *sitABCD* duplications (CRE), *oprD* mutations (CRAB), and *vgrG* SNPs (CRPA), which reverted after antibiotic withdrawal. Fitness cost assays revealed unstable growth deficits in 33.3% (2/6) of HR subpopulations, correlating with genetic instability.

**Discussion:**

These findings highlight the clinical significance of CFD-HR, even in susceptible isolates, and underscore the need for improved diagnostic methods to detect HR and monitor cross-resistance, offering critical insights for regions transitioning to CFD implementation.

## Introduction

Antibiotic resistance caused by carbapenem-resistant (CR) bacteria represents a significant global healthcare crisis, leading to treatment failures and increased hospitalization costs (Band et al., 2019; Behzadi et al., 2022). In recent years, arbapenem-resistant *Enterobacterales* (CRE) and carbapenem-resistant *A. baumannii* (CRAB) have been designated as critical priority pathogens by the World Health Organization (WHO), while carbapenem-resistant *P. aeruginosa* (CRPA) has been categorized in the high priority group (World Health Organization, 2024; Malik and Kaminski et al., 2020). These classifications underscore the urgent public health threat posed by these multidrug-resistant organisms, particularly given the severely limited therapeutic options currently available for managing infections caused by carbapenem-resistant Gram-negative pathogens. It is concerning that even the antibiotics considered the last line of defense, namely tigecycline and polymyxin, have demonstrated emerging resistance (Shields et al., 2020). Moreover, novel synthetic *β*-lactamase inhibitor combinations such as imipenem/relebactam and meropenem/vaborbactam, which were demonstrated efficacy against several multidrug resistance (MDR) Gram-negative bacteria, have been reported to exhibit reduced susceptibility towards certain carbapenem-resistant strains (Zhang et al., 2020; Maraki et al., 2022; Zeng et al., 2022). Cefiderocol (CFD) represents a novel siderophore cephalosporin with potent activity against multidrug-resistant bacteria that can improve the clinical treatment of CR bacterial infections and was approved for marketing in the United States in 2019 (Parsels et al., 2021). Despite most isolates demonstrating susceptibility to CFD in the CREDIBLE-CR trial, a higher mortality rate was observed in the treatment of serious infections caused by CR Gram-negative pathogens accompanied by the emergence of heteroresistance (HR) (Bassetti et al., 2021). The HR phenotype represents a form of bacterial resistance, where subpopulations originating from the same monoclonal source exhibit inconsistent susceptibility to the same antimicrobial agent. This occurs as cells revert back to a decreased antibiotic-sensitive state when the antibiotic pressure diminishes (Dewachter et al., 2019). The presence of an HR subpopulation of bacteria can result in rapid replication in the presence of an antibiotic, while other susceptible bacteria cells are eliminated, may lead to failure of antibiotic treatment and even induce bacterial resistance to drugs. Although epidemiological data have suggested that CFD-HR bacteria might be associated with treatment failure, experimental verification has been lacking (Choby et al., 2021). In China, the prevalence of CR Gram-negative bacteria is becoming an increasingly significant public health issue (Wu et al., 2024).

Reports of HR to antibiotics are on the rise, (Li Y. et al., 2024; Li Y. T. et al., 2024; Stracquadanio et al., 2022) highlighting the critical need for the development or introduction of new antibiotics to effectively combat these resistant strains. At present, CFD has not been incorporated into clinical practice in China, leading to a scarcity of data on the prevalence of HR to CFD. To address this gap, our study focused on CR and extended-spectrum *β*-lactamase (ESBL) producing strains from two major hospitals, identifying instances of CFD-HR and thereby contributing valuable data to the limited existing dataset on CFD-HR prevalence in China. The clinical implications of CFD-HR, especially concerning treatment failure, are still a matter of debate, with findings that remain inconclusive. Since CFD has not yet been officially integrated into clinical protocols in China, our study proposes to use mice models to further investigate the *in vivo* treatment outcomes associated with CFD. Moreover, we conducted adaptive cost experiments and employed whole-genome sequencing (WGS) to preliminarily explore the underlying mechanisms of CFD-HR. These efforts are aimed at providing a foundational understanding of the genetic and phenotypic factors that contribute to CFD-HR, thereby laying the groundwork for future research and potential clinical interventions.

## Materials and methods

### Bacterial strains, growth conditions and plasmids

A total of 407 clinical isolates collected between 2018 and 2022 were included in this study. Among these isolates, 60 strains were obtained from blood samples taken at the Second Affiliated Hospital of Guangzhou University of Chinese Medicine. Among these, CRAB, CRE, and CRPA accounted for 48.3% (29/60), 26.7% (16/60), and 25.0% (15/60), respectively. The remaining 347 isolates were obtained from various specimens at the Affiliated Qingyuan Hospital (Qingyuan People’s Hospital), Guangzhou Medical University. These specimens included sputum, urine, blood, ascites, bile, wound secretions, lavage fluid, cerebrospinal fluid, and throat swabs, accounting for 34.9% (121/347), 5.4% (88/347), 20.7% (72/347), 10.1% (35/347), 3.5% (12/347), 2.9% (10/347), 2.0% (7/347), 0.3% (1/347), and 0.3% (1/347), respectively. Among the isolates from the Qingyuan People’s Hospital, the distribution of strains was as follows: CRAB, CRE, CRPA, *E. coli* produced extended-spectrum *β*-lactamase (eco-ESBL), and *P. aeruginosa* produced extended-spectrum β-lactamase (pae-ESBL) accounted for 18.2% (63/347), 7.5% (26/347), 20.5% (71/347), 51.0% (177/347), and 2.9% (10/347), respectively. The isolates were stored at-70°C and cultured on blood agar plates before conducting the experiments. For broth cultures, the bacteria were cultured at 37°C with shaking at 210 rpm.

Strains were identified as CR or ESBL Gram-negative bacteria according to the Clinical and Laboratory Standards Institute (CLSI) M100 criterial (CLSI, 2023). Quality control strains were purchased from the Quality Control Center of the Clinical Laboratory of Guangdong Province.

### Plasmid construction

For the construction of the *vgrG*-expressing plasmid, the coding sequences of *vgrG* were cloned into the EcoRI site of the *pROp200* plasmid using the Seamless Cloning Kits (Sangon Biotech, Shanghai Co., Ltd), and the NEBbuffer Kits (New England Biolabs Inc.) were used to generate vectors named *pROp200-vgrG*. The resulting plasmid was then transferred to the *PAO1* strain, and the correctness of the cloning was confirmed through PCR and sequencing. The parental *PAO1* strain harboring the empty *pROp200* plasmid served as the empty vector control. All plasmids and primers used for overexpression are listed in Supplementary Tables S1, S2.

### Reagents and antibiotics

Mueller-Hinton agar (MHA; BD Oxoid), LB broth (LB; BD Hopebio), and cation-adjusted Mueller-Hinton broth (CAMHB; BD Hopebio) were used in this study. For CFD experiments, iron-depleted cation-adjusted Mueller-Hinton broth (ID-CAMHB) was used, and it was prepared following the CLSI guidelines (CLSI, 2023). The gradient antibiotic plates for population analysis profile (PAP) test were prepared using MHA with different dilutions of CFD at multiple ratios.

The CFD stock solution used in the experiments was prepared by creating a 25 mg/mL stock solution of CFD powder (BD Absin) in dimethyl sulfoxide (DMSO).

### PAPs

The HR of clinical isolates was determined using PAPs tests following a previously published protocol with some modifications (Band et al., 2019). Overnight cultures of the clinical isolates were prepared by inoculating a single colony from a frozen stock into 3 mL of LB broth. A 10 μL sample of the bacterial solution was then transferred to 2 mL of ID-CAMHB and incubated at 37°C with shaking at 210 rpm until it reached the logarithmic growth phase. The concentration of the bacterial solution was adjusted to 10^7 CFU/mL using ID-CAMHB. Subsequently, 20 μL of the bacterial solution was evenly spread on PAP gradient antibiotic plates. The plates were then incubated at 37°C for 18 to 24 h. After incubation, the number of colonies on each plate was enumerated. The data obtained from the colony counts and the corresponding antibiotic concentrations were analyzed using GraphPad Prism 9 software. HR refers to the presence of a subpopulation of cells capable of proliferating at concentrations of the antibiotic at least eightfold higher than the maximum level that does not impede the replication of the dominant population (Andersson et al., 2019).

### Disk diffusion for antimicrobial susceptibility testing

A single colony of the bacterial isolate was selected and inoculated in an LB broth medium for enrichment culture. After overnight enrichment at 37°C, 10 μL of the bacterial solution was added to 2 mL of ID-CAMHB to inoculate to the logarithmic growth phase. The concentration of the bacterial solution was adjusted to 0.5 McFarland standard using ID-CAMHB, spread on MH agar plates using a sterile cotton-tipped applicator. A disk containing 30ug CFD (Liofilchem) was placed in the center of the plates and incubated at 37°C for 18 h. The zone of inhibition was measured. Results were classified for each species breakpoints according to CLSI guidelines. *E. coli* ATCC 25922 and *P. aeruginosa* ATCC 27853 served as quality control strains in the test.

### Instability test of CFD heteroresistance

**Figure 1:**
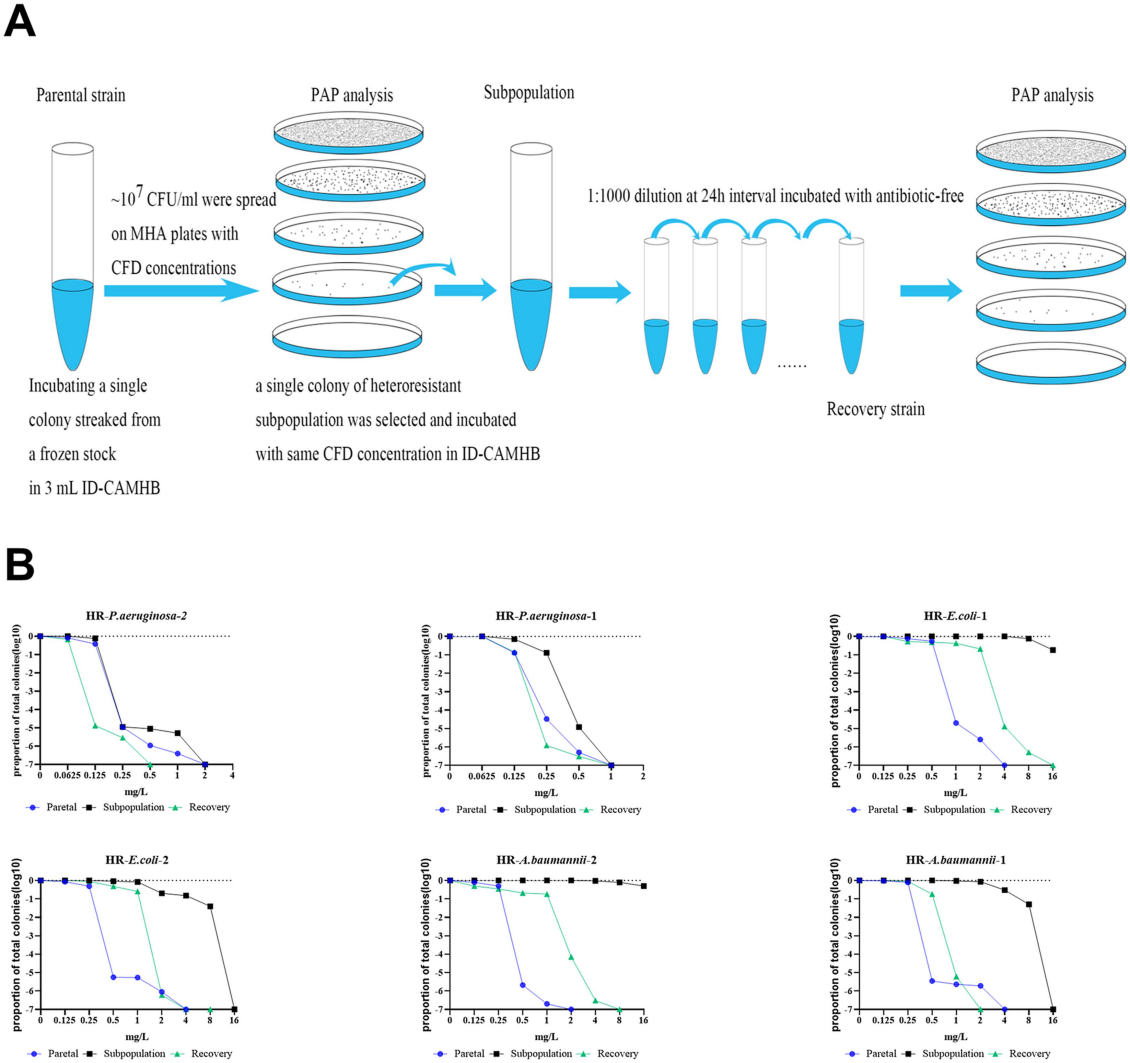
The resistance level against CFD of subpopulations from HR isolates was unstable. **(A)**, A schematic outline of the methods used to determine the instability of HR and the genetic basis of unstable resistance to CFD. **(B)**, After serial passages in antibiotic-free medium, resistant subpopulations reverted to the heterogeneous resistance phenotype showed by the parental bacteria. The vertical axis represents the proportion of bacteria growing at the tested CFD concentrations, normalized to the population in the absence of drugs.

Following confirmation of the HR strain by PAP testing, a single colony was isolated from PAP plates containing the maximum antibiotic concentration at which a subpopulation demonstrated viable growth (Figure 1A). The selected HR colony was transferred into 3 mL of ID-CAMHB medium supplemented with CFD at a concentration equivalent to the PAP plate formulation. The cultures were incubated at 37°C with shaking at 210 rpm for 10 successive passages. During each passage, 3 μL of the bacterial solution was transferred into fresh 3 mL of antibiotics-free ID-CAMHB every 24 h, and this process was performed for a total of 10 passages to obtain the recovery bacteria. The parental bacteria, HR subpopulation and recovery bacteria were then stored at-70°C with 10% glycerol for further tests, such as HR phenotype detection, WGS, and fitness cost measurements. The stability test procedure is illustrated in Figure 1A. PAP was performed on both the resistant subpopulation and the recovery strain. If the resistant phenotype of the recovery strain reverted to that of the original parent isolates, the resistance of HR was considered unstable.

### Growth rates measurements

To analyze the growth rates, a Multifunctional Absorbance Reader (INFINITE 200 PRO, Tecan Austria GmbH) was used. Single colonies of the parental strain, subpopulations, and recovery strain were selected and inoculated in 3 mL of ID-CAMHB medium without antibiotics, except for the subpopulation cultured with CFD at the same concentration used during PAP-mediated the subpopulation selection. After overnight enrichment, 3 μL of the bacterial solution was added to 3 mL of Luria-Bertani (LB) medium and incubated for 8 h at 37°C with shaking at 210 rpm for secondary enrichment. The bacterial culture was then diluted 1:100 with LB medium and added to a 96-well plate with 200 μL per well, and three repeated wells were set for each sample. The optical density at 600 nm (OD600) values was measured every 15 min for 24 h in the Absorbance Reader, with cultures shaken between measurements. The absorbance values between 0.2 and 0.8, representing the exponential growth phase, were used to calculate the maximum growth rate. The experiment was repeated at least three times by picking different colonies on the same plate to ensure the reliability of the results (Band et al., 2019).

### Illumina WGS

In the instability test of CFD-HR, single colonies of parental and recovered isolates were cultured overnight in 3 mL of ID-CAMHB, absent of CFD. A single HR subpopulation colony was inoculated into 3 mL ID-CAMHB containing CFD at the same concentration used in PAP plates and incubated overnight, and bacterial cultures from the mid-exponential growth phase were collected to extract genomic DNA using the TIANamp bacteria DNA kit (Tiangen Biotech, Beijing, China), in accordance with the manufacturer’s guidelines. The extracted DNA was tested using agarose gel electrophoresis for quality assessment and quantified using the Qubit® 2.0 Fluorometer. A total of 1 μg DNA per sample was employed for DNA sample preparations. The whole genomes of the parental strain, subpopulation, and recovery bacteria were sequenced by the Illumina NovaSeq PE150 platforms (Novogene Bioinformatics Technology Co., Ltd. Beijing, China). The original high-throughput sequencing data were converted into raw sequenced reads by CASAVA base calling and stored in FASTQ (fq) format, which encompassed sequencing information and the corresponding read sequencing quality data. The sequenced data were filtered, and the sequence of Adapter and low-quality data were excised, resulting in clean data for subsequent analysis. Specific processing steps are as follows: (1) Eliminate reads whose low-quality nucleotides (*Q*-value ≤ 20) exceeding certain threshold (40 bp by default). (2) Eliminate reads which contain N nucleotides exceeding certain threshold (10 bp by default). (3) Eliminate reads whose overlap with adapter exceeding certain threshold (15 bp by default). (4) If the sample exist contamination, Blast will performed against with host database to filter reads probably generated from the host (https://www.ncbi.nlm.nih.gov/bioproject; BioProject ID PRJNA1067195).

### Mouse model of peritonitis infection

**Figure 2:**
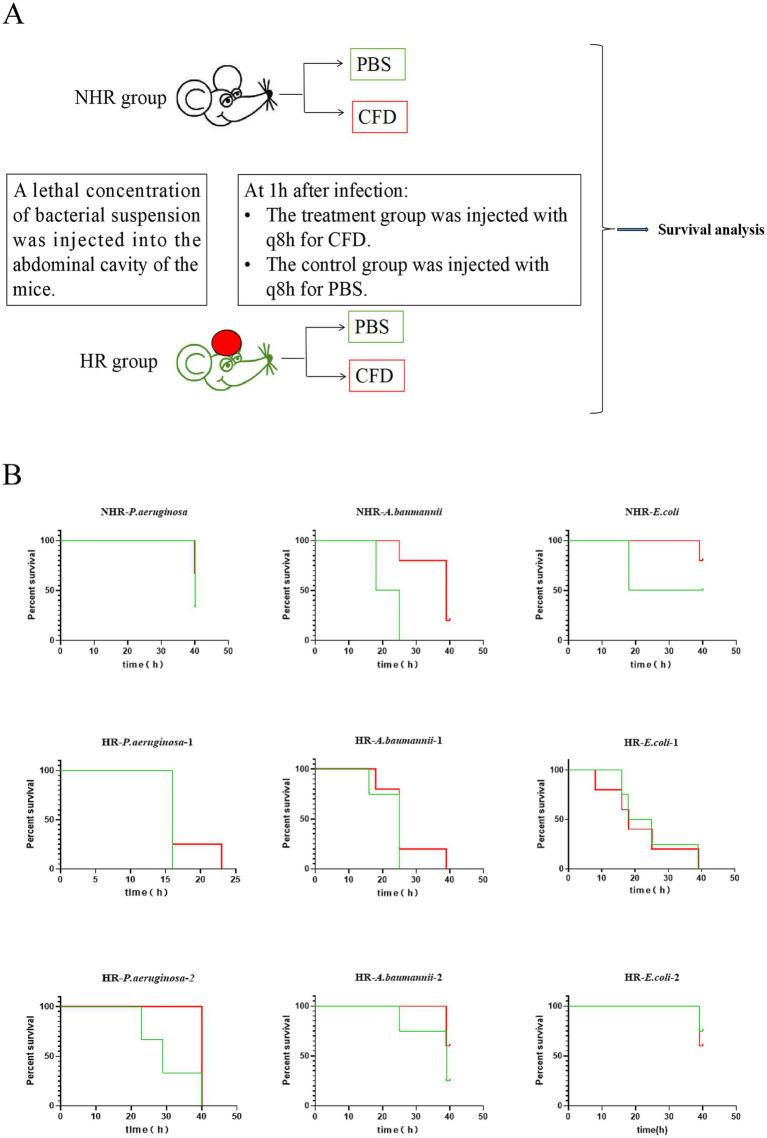
HR leads to *in vivo* CFD treatment failure. **(A)**, A schematic outline of the mouse infection model. Strains in both *in vivo* and *in vitro* (Time-kill assay) were derived from same colonies. Mice were injected with a lethal concentration of bacteria and then randomly divided into a treatment group (CFD group) and a control group (PBS group) Mice in the CFD group received CFD injections at 8-h intervals (q8h) starting 1 h after infection, while mice in the PBS group received PBS injections. **(B)**, In groups of mice with NHR bacterial infection, all mice in the CFD group (red line) had higher survival rates compared to those in the PBS group (green line). Among the groups of mice infected with HR bacteria, mice showed little different survival rates in the CFD group compared to the PBS group.

Male C57BL/6 mice weighing 18–25 g and aged 6–8 weeks were obtained from SpePharm (Beijing) Biotechnology Co., Ltd. (animal license number: SCXK 2019–0010). They were injected intraperitoneally with a minimal lethal dose of bacteria (1*10^8^), then randomly divided into a treatment group and a control group, with 3 to 5 mice in each group. In the treatment group, 100 μL of CFD (2 μg/μL) was injected intraperitoneally every 8 h, starting 1 h after bacterial infection. In the control group, 100 μL of phosphate-buffered saline (PBS) was injected intraperitoneally every 8 h. The survival of the mice was recorded for 40 h after infection. The detailed experimental protocol is illustrated in Figure 2. Peritoneal lavage fluids were collected 24 h post-infection to count the subpopulation and total bacterial colonies. 10μl peritoneal fluid and pre-injection bacterial were spread on MH plate containing CFD (the concentration equivalent to the PAP plate derived for the HR subpopulation) and the antibiotic-free MH plate, and overnight cultured in 37°C. The number of colonies on the plate was counted. The HR subpopulation is obtained by counting the number of colonies on the MH plate containing CFD. The proportion of HR subpopulation was calculated by dividing the number of HR subpopulation by the total number of bacteria obtained by counting the colonies on the antibiotic-free MH plate. All experiments were conducted in compliance with the approved protocols and guidelines of the Animal Experiment Center of Qingyuan People’s Hospital.

### Time-kill assay

A single colony of the bacterial strain was selected and inoculated in an LB broth medium. After overnight incubation at 37°C and 210 rpm, 10μl of the bacterial solution was added to 2 mL of fresh LB broth medium for further enrichment to the logarithmic growth phase. The concentration of the bacterial solution was adjusted to 10^6^ CFU/mL, and 3 mL of the bacterial solution was distributed into four sterilized glass test tubes. One test tube was kept without antibiotics, while the other three test tubes were supplemented with CFD at final concentrations of 4 mg/L, 8 mg/L and 16 mg/L, respectively. The four test tubes were then incubated at 37°C and 210 rpm for 11 h, following by centrifugation, the supernatant was removed and the bacteria were collected and the culture was continued for up to 24 h in a new medium containing the above concentration of antibiotics. At different time points (0 h, 4 h, 6 h, 8 h, 10 h, 12 h, and 24 h), 10μl of bacterial solution was collected from each test tube, and multiple dilutions were prepared with PBS at a 10-fold dilution factor. 10μl of each diluted bacterial solution was spread onto nutrient agar plates and incubated at 37°C overnight. The number of bacterial colonies on each plate was then counted.

### Statistical analysis

Data from multiple independent experiments are presented as the mean ± standard deviation (SD). Statistical differences between two groups were analyzed using Student’s t-test, while one-way ANOVA was used for comparisons involving more than two groups. GraphPad Prism version 9 software (GraphPad Software, Inc., San Diego, CA, United States) was used for all statistical analyses. Statistical significance is represented as follows: * for *p*-values between 0.01 and 0.05, ** for *p*-values less than 0.01, and *** for *p*-values less than 0.001.

## Results

### Prevalence of CFD-HR in clinical carbapenem-resistant gram-negative isolates

**Figure 3:**
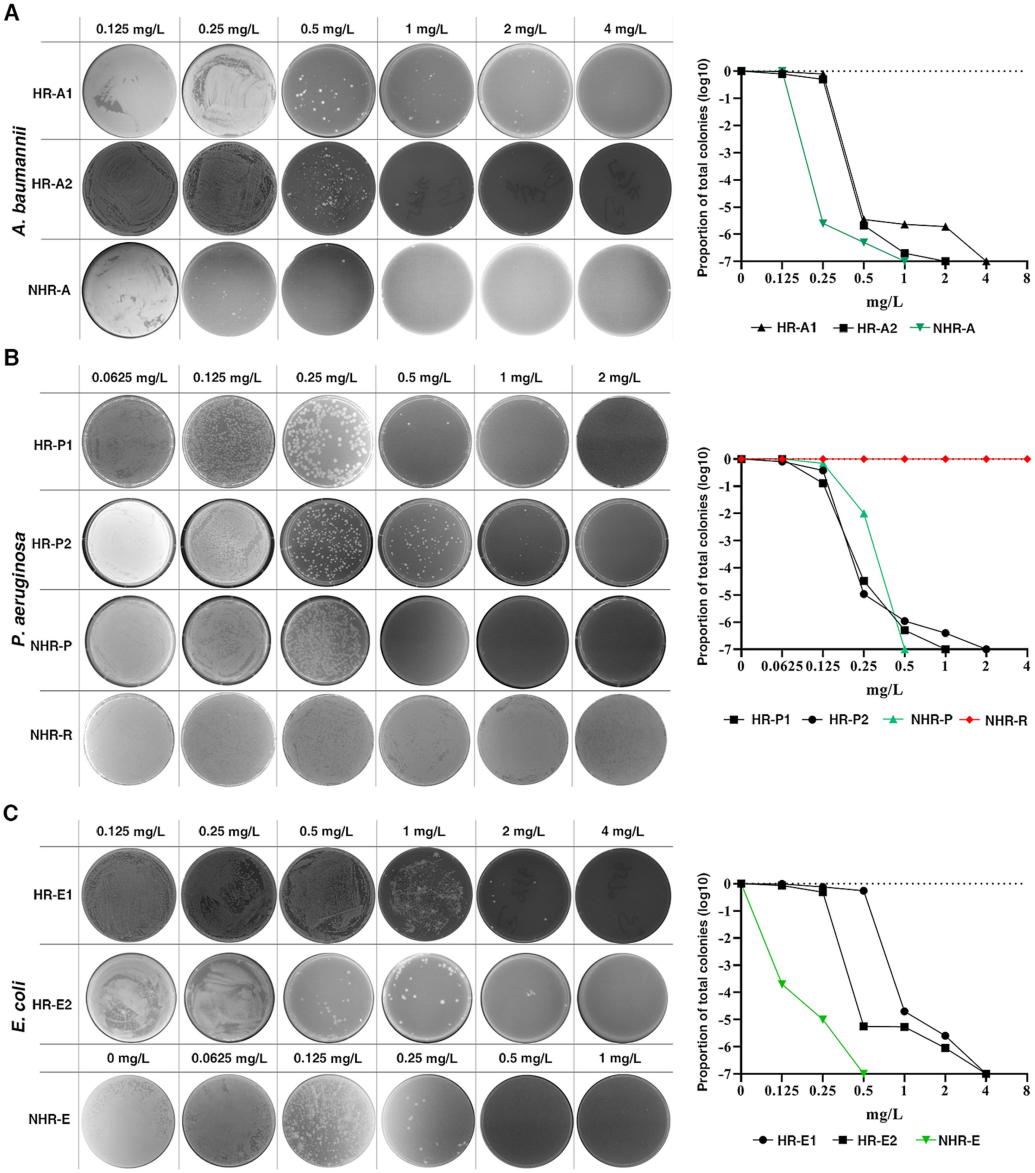
Prevalence of CFD-HR in clinical isolates of the pathogens. HR was observed in carbapenem-resistant *A. baumannii* **(A)**, *P. aeruginosa* **(B)** and *E. coli* **(C)**. Representative isolates were plated on a series of CFD concentrations with population analysis profiles (PAPs). The proportion of total colonies was calculated compared to growth on the drug-free plates. The black line, green line and red line represent HR bacteria, non-HR bacteria and resistant bacteria, respectively. HR was defined as the presence of a subpopulation of cells capable of growing at concentrations of the antibiotic at least eightfold higher than the highest concentration that does not affect the replication of the dominant population.

To investigate the prevalence of CFD-HR among carbapenem-resistant bacteria, we performed population analysis profiles (PAPs) on 407 clinical isolates, including carbapenem-resistant *A. baumannii* (CRAB, *n* = 92), carbapenem-resistant *P. aeruginosa* (CRPA, *n* = 86), *P. aeruginosa* produced extended-spectrum *β*-lactamase (pa-ESBL, *n* = 10), carbapenem-resistant *E.coli* (CRE, *n* = 42) and *E. coli* produced extended-spectrum β-lactamase (eco-ESBL, *n* = 177), from two clinical centers in Southern China. Among these isolates, 16 (17.4%) CRAB, 24 (27.9%) CRPA, 10 (23.8%) CRE, 1 (10.0%) pae-ESBL and 8 (4.5%) eco-ESBL were identified as HR to CFD. The HR rate of CR bacteria (≥17.4%) was higher than that of ESBL-producing bacteria (≤10.0%) (Figure 3, Table 1). The susceptibility of HR-positive isolates was detected by the disk diffusion method according to CLSI guidelines (CLSI, 2023). Among the HR-positive isolates, 72.9% (43/59) were classified as susceptible. However, there were a few exceptions, where HR strains were classified as intermediate or resistance (Table 2).

**Table 1:** The distribution of HR bacteria.

Bacteria	Isolates	HR isolates	HR rates
CRPA	86	24	27.9%
CRE	42	10	23.8%
CRAB	92	16	17.4%
pae-ESBL	10	1	10.0%
eco-ESBL	177	8	4.5%
Total	407	59	14.5%

CRPA, carbapenem-resistant *Pseudomonas aeruginosa*; CRE, carbapenem-resistant *Enterobacterale*; CRAB, carbapenem-resistant *Acinetobacter baumannii*; pae-ESBL, *P. aeruginosa* produced extended-spectrum β-lactamase; eco-ESBL, *Escherichia coli* produced extended-spectrum β-lactamase; HR, heteroresistant.

**Table 2:** The distribution of heteroresistant bacteria in the MIC range.

MICs classify	NHR bacteria	HR bacteria	Distribution of heteroresistant bacteria	Distribution of non-heteroresistant bacteria
Resistant	27	7	11.9% (7/59)	7.8%(27/348)
Intermediate	57	9	15.3% (9/59)	16.4%(57/348)
Susceptible	264	43	72.9% (43/59)	75.9(264/348)
Total	348	59	100% (59/59)	100%(348/348)

HR, heteroresistant; NHR, non- heteroresistant; MIC, Minimum Inhibitory Concentration.

### HR was associated with treatment failure following CFD administration in both *in vitro* and *in vivo* models

**Figure 4:**
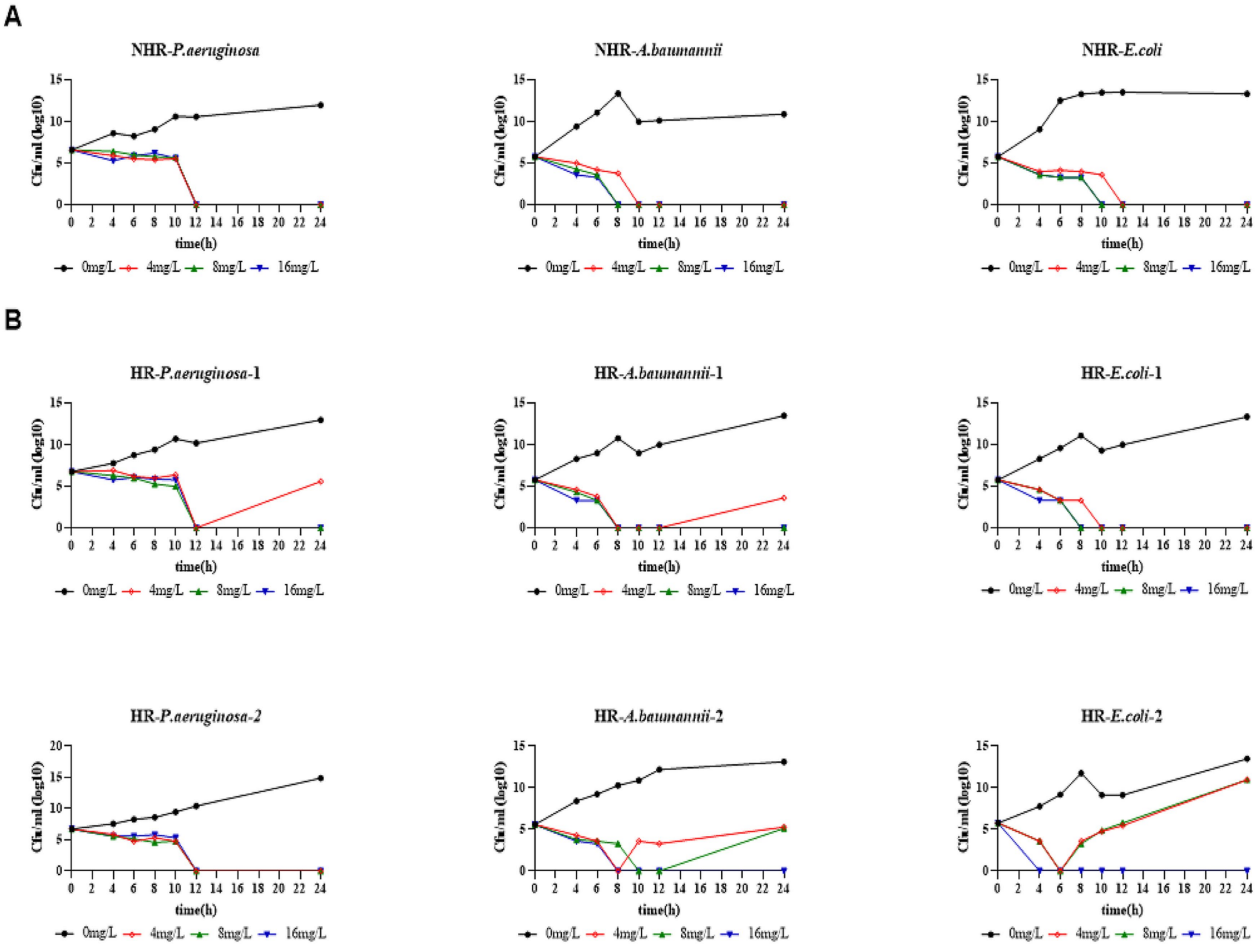
HR bacteria required higher bactericidal concentrations than non-HR bacteria. **(A)**, Time-kill assay in non-HR bacteria. **(B)**, Time-kill assay in HR bacteria. All strains in the bactericidal curve were sensitive strains (corresponding to MIC≤4mg/L). The indicated concentrations of CFD were supplemented at 0 h and re-added after 11 h to maintain a constant antibiotic concentration in each tube.

The effect of CFD’s bactericidal ability *in vitro* was evaluated using the Time-kill assay (TKA). Nine isolates were selected, including 2 HR strains each of CRAB, CRPA, and CRE, and 1 non-HR strain each of CRAB, CRPA, and CRE, all of which were classified as susceptible in disk diffusion with corresponding MIC ≤4 mg/L determined by the microdilution method. For the non-HR (NHR) isolates, treatment with 4 mg/L of CFD resulted in sustained initial killing against all CRPA (NHR-*P. aeruginosa*), CRAB (NHR-*A. baumannii*), and CRE (NHR-*E. coli*) (Figure 4A). However, upon treating the HR isolates with 4 mg/L of CFD, 2 of 6 (HR-*P. aeruginosa-*2 and HR-*E. coli-*1) were killed within 12 h and did not regrow within 24 h, while the remaining HR isolates, including CRPA (HR-*P. aeruginosa-*1), CRAB (HR-*A. baumannii-*1, HR-*A. baumannii-*2) and CRE (HR-*E. coli-*2), regrow after 12 h following treatment with either 4 mg/L or 8 mg/L of CFD, even though the bacterial count decreased by more than 3 log values prior to 12 h (Figure 4B). These results indicate that higher CFD concentrations than the susceptible breakpoint are required to effectively eliminate HR bacteria compared to NHR bacteria, even when both are classified as susceptible.

To investigate whether HR affects the outcome of CFD treatment during an *in vivo* infection, we constructed a mouse model of peritonitis and evaluated its relevance on therapy outcomes (Figure 2A). C57BL/6 mice were intraperitoneally infected with a lethal dose of CFD-susceptible CRAB, CRPA and CRE isolates which were same as strains in *in vitro*. The strains were with or without HR (HR + and HR-, respectively). In the non-HR group infected with CRPA (NHR-*P. aeruginosa*), CRAB (NHR-*A. baumannii*) and CRE (NHR-*E. coli*), the treatment with CFD resulted in significantly increased survival rates (100%, 3/3) and compared to the control group treated with PBS. Conversely, in the HR group, no significant differences in survival rates (16.7%, 1/6) or survival time were observed between the treatment with CFD and PBS, except for one CRAB strain (Figure 2B).

The frequency of the resistant subpopulation in both the non-HR (NHR-*P. aeruginosa*) and HR (HR-*P. aeruginosa-*1) isolates infected mice was subsequently calculated. Peritoneal lavage fluids were collected 24 h post-infection to count the subpopulation and total bacterial colonies. For isolate HR-*P. aeruginosa-*1, the frequency of the resistant subpopulation notably increased after 24 h of *in vivo* infection compared to the pre-infected inoculum, possibly due to cross-resistance to host innate immune antimicrobials and reactive oxygen species, as reported previously. More importantly, the frequency of resistant subpopulation further increased after treatment with CFD. Interestingly, the frequency of the resistant subpopulation of isolate NHR-*P. aeruginosa* from mice peritoneal lavage fluids decreased compared to the inoculum, and this decrease was enhanced by treatment with CFD (Supplementary Figure S1).

These compelling *in vitro* and *in vivo* findings provide strong evidence that HR may contribute to the failure of CFD treatment for CRAB, CRPA and CRE infections.

### Heteroresistance to CFD is unstable and transient

HR represents a form of resistance wherein a fraction of the bacterial population exhibits a transient increase in antibiotic sensitivity that is unstable and reversible (Dewachter et al., 2019). Here, we conducted PAPs analysis to assess the phenotypic stability of resistant clones from the two subpopulations of HR CRPA, CRAB and CRE on the original isolates (parental strain, Ori), the resistant subpopulations from plates with the highest CFD concentration of PAPs (Subpopulation), and the resistant subpopulations after 10 generations in the absence of antibiotic pressure (Recovery strain) (Figure 1A). Upon comparing the original isolate to the resistant subpopulations (Sub), we observed an increase in both the highest CFD concentration on which the subpopulation could grow and the quantity of subpopulation colonies present on plates with the same CFD concentration, indicating a higher level of resistance in the subpopulations. However, for the reverse strains, there was complete reversion for about half the strains, and the other half had a reversion that trended back toward the parent strain but still possessed a higher level of non-susceptibility (Figure 1B).

**Figure 5:**
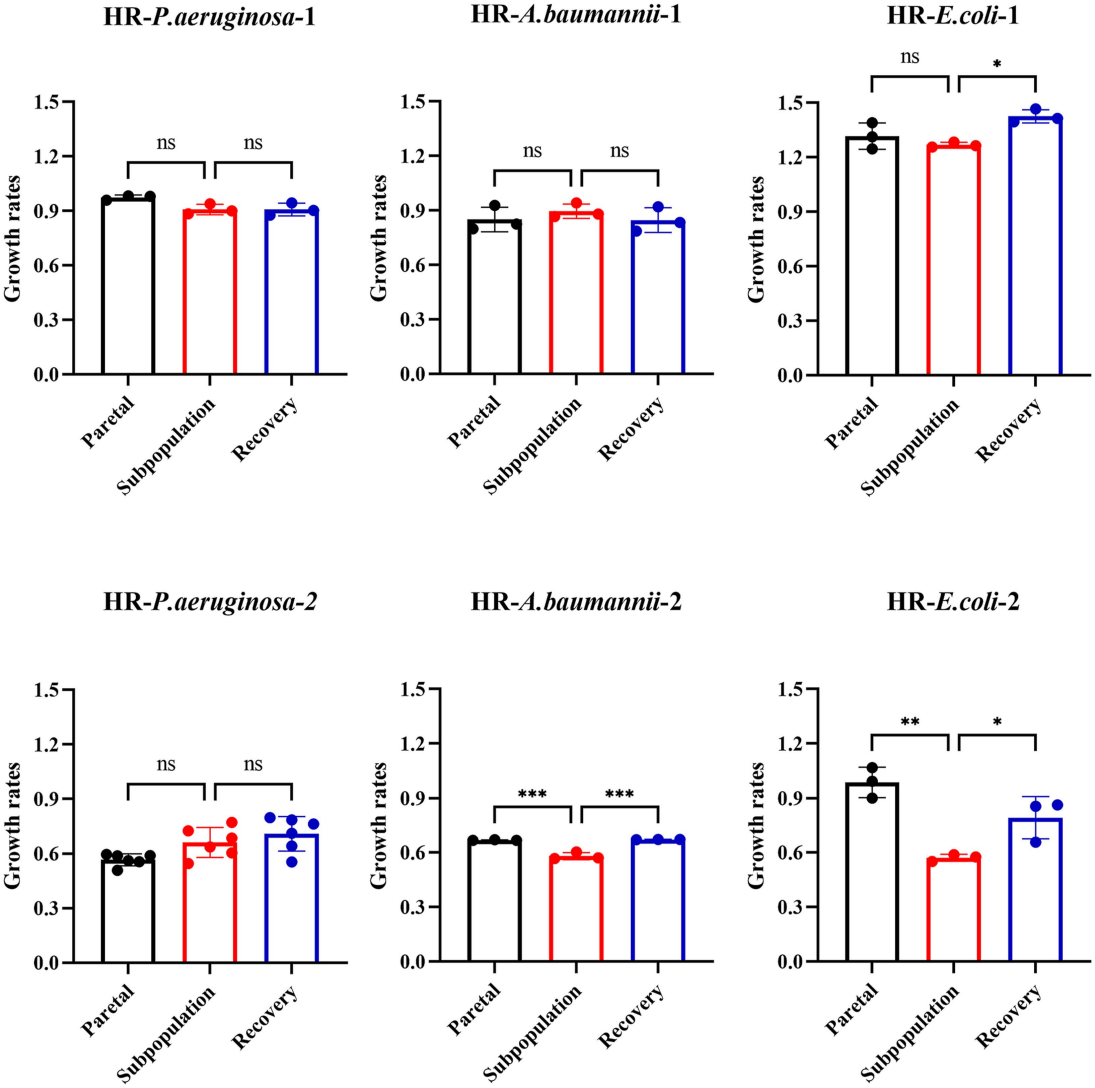
Fitness costs increased partially in subpopulations and reverted to recovery strain without antibiotics. The fitness cost was assessed by measuring the growth rates. OD600 values were recorded every 15 min for 24 h. Growth rates were calculated during the exponential phase, where absorbance values ranged between 0.2 and 0.8.

The fitness cost was assessed by measuring the growth rate of the parental bacteria, subpopulation, and recovery bacteria in the six strains. Among these strains, approximately 33.3% (2 out of 6) of the subpopulations exhibited an increased fitness cost, which was later reverted in the recovery strain. However, no significant differences were observed in the growth rate among the parental strain, subpopulation, and recovery strain for the other four strains (Figure 5).

Taken together, these results indicate that the resistance of subpopulations was unstable and partially associated with a higher fitness cost.

### Unstable resistance of subpopulation linked to unstable gene mutation

To elucidate the mechanism underlying the unstable HR phenotype, we conducted WGS on the parental strain, subpopulation, and recovery strain of the six strains mentioned above, based on which we identified several genes that were frequently mutated and duplicated, specifically in the HR subpopulation, rather than in the parental or recovered strains. In the subpopulation of CRPA, the most common mutations were single nucleotide polymorphisms (SNPs), accounting for 82.4% (14 out of 17 mutations), with no duplications observed. On the other hand, in the subpopulations of CRE and CRAB, deletions were the most common type of mutation, comprising 69.7% (46 out of 66 mutations) and 62.9% (39 out of 62 mutations), respectively. Duplications accounted for 13.6% (9 out of 66 mutations) in CRE and 9.7% (6 out of 62 mutations) in CRAB (Supplementary Table S3).

**Figure 6:**
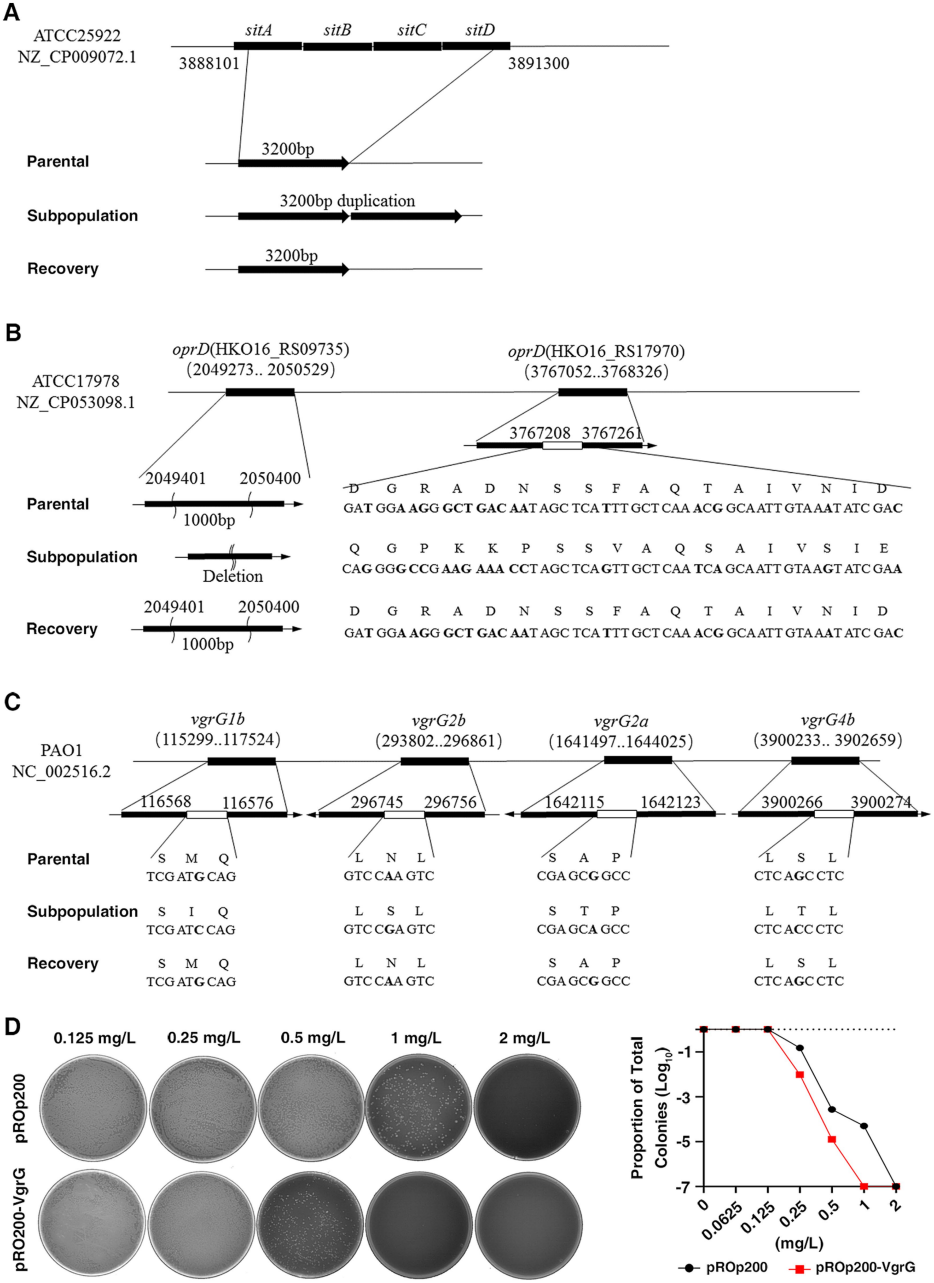
Frequent mutation and duplication were observed in the subpopulation detected by whole-genome sequencing (WGS). **(A)**, In two carbapenem-resistant *Enterobacteriaceae* (HR-*E.coli*-1, HR- *E.coli*-2) strains, mapping the sequencing reads to reference genomes derived from ATCC25922, a common mutation with duplication was detected in the iron uptake gene *sitABCD* in the subpopulation, which was not found in the recovery and parental strains. **(B)**, In the two carbapenem-resistant *A. baumannii* (HR- *A. baumannii-*1, HR- *A. baumannii-*2) strains, mapping the sequencing reads to reference genomes derived from ATCC17978, the gene *oprD* in the subpopulation displayed consistent mutations with deletions, SNPs, and inserts, while no such genic mutations were observed in the parental and recovery strains. **(C)**, In the two carbapenem-resistant *P. aeruginosa* (HR-P1, HR-P2) strains, mapping the sequencing reads to reference genomes derived from PAO1, a common SNP mutation was detected in the *vgrG* gene. **(D)**, The impact of the *vgrG* gene on CFD-HR was confirmed by constructing a *vgrG*-overexpressed strain (*pROp200-VgrG*). The HR tests (PAPs) were performed, and MIC of *vgrG*-overexpressed strain (1 mg/L) was decreased compared to the control (*pROp200*, 2 mg/L).

In the two CRE isolates (HR-*E. coli*-1 and HR- *E. coli*-2), we observed duplication of the iron uptake gene *sitABCD* locus in the subpopulation and lost in recovery strains (Figure 6A). s*itABCD* is known as a TonB-independent Ferrous ion uptake system that facilitates Fe^2+^ across the cytomembrane, which may influence the susceptibility of bacteria against CFD through regulating TonB-dependent transporters (TBDTs). In two CRAB isolates (HR-*A.baumannii-*1, HR-*A. baumannii-*2), we observed mutations, including deletions, SNPs and insertions, in the *oprD* gene only in the subpopulation but not in the parental and recovered strains (Figure 6B). Previous studies have shown an association between reduced expression of porin gene *oprD* with HR (Do and Timsit, 2023). For two CRPA isolates (HR-*P. aeruginosa-*1 and HR-*P. aeruginosa-*2), the subpopulation had a common SNP mutation in the *vgrG* gene, which was not present in the parental or recovery strains (Figure 6C). The role of *vgrG* in antibiotic resistance has been explored in some previous studies (Wang et al., 2018). To further investigate the effect of *vgrG* on CFD-HR, we constructed a *vgrG*-overexpressed strain, and our results indicated that the restored expression of *vgrG* in the resistant subpopulation led to an attenuated resistance level, suggesting that mutations and recovery of *vgrG* could contribute to the unstable HR of *P. aeruginosa* (Figure 6D).

## Discussion

In comparison to *β*-lactam/β-lactamase inhibitor conjugations, carbapenems and advanced-generation cephalosporins, CFD has distinct advantages in regard to its antimicrobial activity against CR Gram-negative bacilli stemming from its unique properties, such as (1) a siderophore-like cell entry mechanism, which allows the generation of a high concentration of the drug at the periplasm, and (2) stability against hydrolysis by nearly all β-lactamases, including both serine- and metallo-β-lactamases, resulting in enhanced antimicrobial activity (Zhanel et al., 2019). In this study, we present the first report of CFD-HR (CRPA, CRAB and CRE) in China, where CFD is not yet widely used in clinical practice. More importantly, our research demonstrated that HR not only led to a diminished bactericidal effect of ERV *in vitro* but also had even more serious implications in an *in vivo* infection model. Among the carbapenem-resistant isolates, there had been a previously reported correlation between CFD-HR and increased all-cause mortality in the CREDIBLE-CR trial, and in this present study, our findings provide further direct evidence that HR contributed to the treatment failure of CFD. Notably, approximately 72.9% (43/59) of HR-positive isolates were classified as susceptible by disk diffusion. Considering that clinicians might turn to CFD as a last-line therapy for infections caused by carbapenem-resistant bacteria, the detection of CFD-HR, which is currently not captured by current clinical laboratory susceptibility testing, calls for the development of more accurate and sensitive diagnostic methods.

PAP test is regarded as the “gold standard” method for detecting HR (Longshaw et al., 2023; Koçer et al., 2024). This technique exposes bacterial populations to progressively increasing concentrations of antibiotics under high cell density conditions, thereby identifying any minority resistant subpopulations that may be overlooked by the lower inoculum sizes used in reference susceptibility tests. Among the 407 clinical isolates we investigated using PAP, the overall prevalence of CFD-HR was found to be 14.5%, which was lower than the reported prevalence in the USA (Choby et al., 2021). It is important to note that CFD is a modified cephalosporin, and its structure and bactericidal mechanism are similar to traditional cephalosporins (Zhanel et al., 2019). A noteworthy observation is the reported cross-resistance among ceftazidime/avibactam, ceftolozane/tazobactam, and CFD (Hobson et al., 2021; Bianco et al., 2022; Poirel et al., 2022). These traditional cephalosporins are widely used in clinical practice in China, and therefore, the observed prevalence of CFD-HR may be influenced by the cross-HR with traditional cephalosporins (Fröhlich et al., 2022; Russo et al., 2022), which highlights the significance of monitoring and understanding the potential impact of cross-resistance on the effectiveness of CFD treatment in clinical settings.

In phase III clinical trials of CFD, there were suggestions of a possible relationship between treatment failure and HR, but direct evidence from systematic trials remained lacking (Choby et al., 2021; Timsit et al., 2022). In this present study, we found that although all isolates used in TKA were sensitive bacteria, dynamic observation of the bactericidal curve showed that only 33.3% (2/6) of HR bacteria could be killed without regrowth by 4 mg/L of CFD (breakpoint of susceptible). Most HR bacteria (66.7%, 4 out of 6) required higher minimum bactericidal concentration (MBC) levels, ranging from 8 mg/L to 16 mg/L, compared to non-HR bacteria. Notably, TKA demonstrated that the CFD killing activity was considerably reduced in the presence of HR bacteria. The findings were consistent with previous reports on the subject (Wang et al., 2022; Mezcord et al., 2023). It has long been established that HR can lead to treatment failure *in vitro* settings. However, the potential for HR to cause treatment failure *in vivo* has remained a subject of considerable debate (Karakonstantis et al., 2022; Shields et al., 2024). In China, CFD had not yet been commercialized, and as a result, the clinical outcomes of these strains could not be directly observed. To elucidate the *in vivo* behavior of HR strains, we conducted animal experiments using mice. Our *in vivo* experiments demonstrated that mice infected with HR bacteria treated with CFD had significantly lower survival rates than those in the NHR group. Thus, these findings demonstrate that while HR is not an absolute determinant of treatment failure, it constitutes an important risk factor for suboptimal therapeutic outcomes. To the best of our knowledge, this study is the first to provide *in vivo* experimental evidence exploring the link between HR and treatment failure of CFD.

Consistent with previous studies (Nicoloff et al., 2019) our results confirmed the transient and unstable resistant level of the HR subpopulation. Genetic analysis of the subpopulation, parental, and recovered strains revealed various gene alterations affecting multiple functions, such as porin, iron uptake, and bacterial secretion systems. Some of these gene mutations have been previously associated with resistance to specific antibiotics. Recent studies have indicated that the deletion of the *vgrG* gene led to increased antimicrobial resistance to ampicillin/sulbactam but decreased resistance to chloramphenicol (Wang et al., 2018). To date, few studies have been reported specifically focusing on this particular gene within the context of research involving CFD-HR. In our present study, we identified subpopulations of *P. aeruginosa* that exhibited a common SNP mutation in the *vgrG* gene. Further experiments demonstrated that overexpression of the *vgrG* gene resulted in reduced resistance to CFD. This suggests that mutations and recovery of the *vgrG* gene may contribute to the instability of HR and the presence of subpopulations in *P. aeruginosa*. As previously discussed, the emergence of subpopulations is a critical determinant in the reduced antibiotic susceptibility observed in HR strains. In this context, the findings of the present study elucidating the role of the *vgrG* gene in HR provided valuable supportive evidence that may aid in deciphering the underlying mechanisms of CFD-HR. In both *A. baumannii* strains (HR-*A. baumannii-*1, HR-*A. baumannii-*2), mutations in the genes HKO16_RS09735 and HKO16_RS17970 related to *oprD* were observed only in the subpopulation, not in the parental or recovery strains. Similar genetic changes, including single nucleotide polymorphisms and insertional elements in *oprD*, have been frequently found in MDR *A. baumannii* strains, and down regulation of *oprD* was observed in MDR and pan-drug-resistant *A. baumannii* clinical strains (Yang et al., 2015; Zhanel et al., 2019; Stracquadanio et al., 2024). More importantly, resistance against CFD was found to be increased in the *oprD*-deficient strains indicating that the mutation of *oprD* in the subpopulation and its recovery in the recovered strain in our study could be involved in the unstable HR of *A. baumannii*.

It has been previously reported that a high prevalence of antibiotic HR in pathogenic bacteria is primarily caused by gene amplification. In our study, we observed the duplication of *sitABCD* genes in the subpopulation of *E. coli* (HR-*E. coli*-1 and HR- *E. coli*-2), which was distinct from the parental and recovered strains. *sitABCD* is a TonB-independent transporter responsible for the uptake of ferrous iron. The involvement of the *sitABCD* gene in the potential mechanism of HR is related to the uptake of soluble Fe^2+^. Specifically, the *sitABCD*-facilitated uptake of Fe^2+^ may lead to the downregulation of TonB-dependent transporters (TBDTs), resulting in decreased susceptibility to CFD (Carpenter and Payne, 2014; Luscher et al., 2018). Hence, the reversible amplification and loss of *sitABCD* may contribute to the unstable HR of *E. coli* and impact the effectiveness of CFD treatment. Nonetheless, while our current findings provide valuable insights, additional confirmatory experiments are warranted in the future to fully elucidate the underlying mechanisms and validate the observed phenomena. This will be essential to strengthen the conclusions drawn from our study and to pave the way for further advancements in this area of research.

Most resistance mutations in bacteria carry a fitness cost. As many antibiotics target important cellular processes, resistance to them may disrupt those processes or impose a large energetic burden that reduces the competitive power of the susceptible strain, leading to a change in fitness costs that manifests as a reduction (Melnyk et al., 2015). Our study showed that only one *E. coli* (HR- *E.coli*-2) and one *A. baumannii* (HR- *A. baumannii-*2) among the six isolates exhibited an increase in the fitness of the subpopulation compared to that of the parent bacteria. However, this increased fitness was reverted to the level of the parental strain after 10 passages, coinciding with the loss of gene *sitABCD* duplication or the frequent mutation of gene *oprD*. In contrast, no significant growth variability was observed in the subpopulations of the other four HR strains. Nonetheless, the fitness of certain subpopulations was observed to increase, albeit in an unstable manner. This instability may contribute to the poor correlation between clinical treatment outcomes and the detection of HR. Therefore, further multidimensional experiments are required to elucidate the underlying mechanisms of HR instability.

The findings of our study reveal that CFD-HR bacteria are prevalent in clinical settings, even in regions where CFD has not yet been implemented. This observation underscores the potential for HR to emerge and persist independently of direct CFD exposure, highlighting the importance of proactive surveillance and targeted interventions to mitigate the spread of such resistant strains. It is worth noting that although our study demonstrates that CFD-HR is widespread among clinical carbapenem-resistant Gram-negative strains. However, there may be some limitations in the generalizability of the study because our prevalence survey population included only two hospitals. Evidence from *in vivo* and *in vitro* experiments strongly suggests that HR bacteria, even if classified as susceptible, can lead to the failure of CFD treatment due to the presence of an enhanced resistant subpopulation. However, this enhanced resistance in the HR subpopulation is unstable and may be associated with a fitness cost. The instability of resistance and the associated fitness costs within subpopulations may contribute to the observed variability in clinical outcomes. The underlying genetic mechanisms driving these phenomena may involve genetic duplication or the frequent mutation. In our study, WGS analyses identified several genes that appear to be related to HR. These findings provide valuable insights into the potential genetic determinants of resistance instability. However, it is important to note that the identification of these genes through WGS is only the first step in unraveling the complete picture. Further validation studies are essential to confirm the functional roles of these genes in HR and to elucidate the precise mechanisms through which they contribute to resistance instability. In order to further explore the specific mechanisms of bacterial heterogeneous resistance, we are still working on genes other than *vgrG* that may affect bacterial heterogeneous resistance, which is very important for the treatment of clinically resistant bacteria.

## Data Availability

The datasets presented in this study can be found in online repositories. The names of the repository/repositories and accession number(s) can be found in the article/Supplementary material.
