# Supplementary material for: Carbapenem-resistant Gram-negative bacteria exhibiting clinically undetected cefiderocol heteroresistance leads to treatment failure in a murine model of infection
Source: Front Microbiol. 2025 May 9;16:1496514. doi: 10.3389/fmicb.2025.1496514 (PMC12098276; doi:10.3389/fmicb.2025.1496514)
Supplement: Supplementary file 1 [file Data_Sheet_1.docx]

**Supplementary**

**Plasmids construction**

For construction of *vgrG*-expressing plasmid, *vgrG* coding region was amplified by PCR from PAO1 chromosomal DNA. Amplified fragments were purified by gel extraction kit (Sangon Biotech, Shanghai, China), then digested with EcoRI-HF (New England Biolabs Inc), and cloned into the corresponding sites of plasmid prop200.

**The rates of heteroresistant (HR) subpopulation counting**

Six to eight weeks old mice (weighing 18~25 g) were injected intraperitoneally with minimal lethal dose of bacteria were randomly divided into treatment group (CFD group) and control group (PBS group). Mice in the treatment groups were injected with cefiderocol (CFD) 100ul (2ug/ul) one hour after bacterial injection, while mice in the control groups were injected with PBS 100ul. After 8h, the mice were killed by carbon dioxide asphyxiation and 1ml of PBS was injected into the abdominal cavity of the mice using a syringe for rinsing, then the abdominal fluid was withdrawn. 10ul peritoneal fluid and pre-injection bacterial (pre-injection was constantly stored in 4℃ before spread on plates) fluid were spread on MH plate containing CFD (the concentration of CFD is 0.5×MIC) and the antibiotic-free MH plate, and overnight cultured in 37℃. The number of colonies on the plate was counted. The HR subpopulation is obtained by counting the number of colonies on the MH plate containing CFD. The proportion of HR subpopulation was calculated by dividing the number of HR subpopulation by the total number of bacteria obtained by counting the colonies on the antibiotic-free MH plate.

**
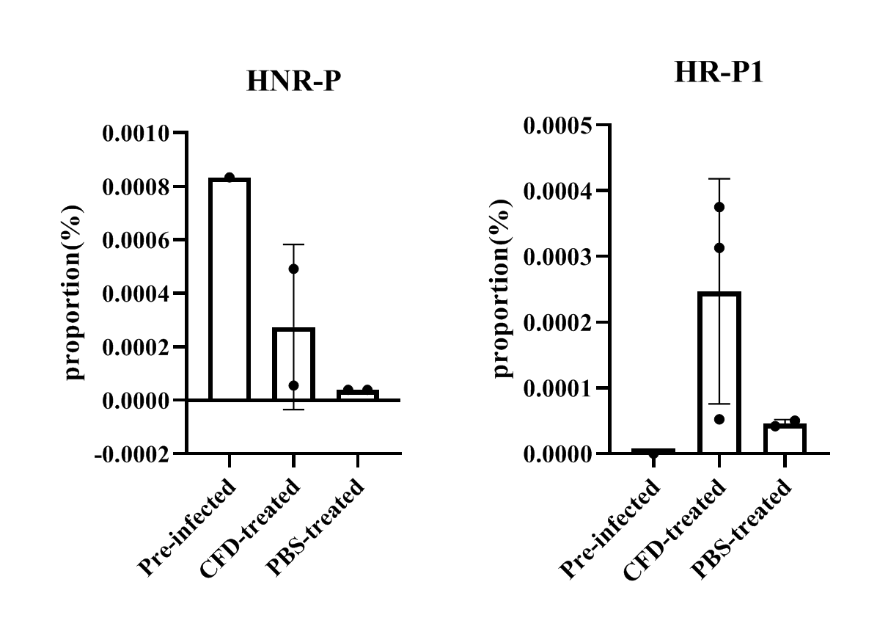
**

**Fig. S1 HR subpopulation rates in abdominal cavity of mice.**

In the HR bacteria injection group (HR-P1), the proportion of subpopulations after CFD treatment (CFD-treated) was higher than that before treatment (pre-infected). In the NHR bacterial injection group (HNR-P), the proportion of subpopulations after CFD treatment (CFD-treated) was lower than that before treatment (pre-infected). Whereas the increase in proportion of PBS-treated subpopulations was almost negligible. It is indicated the increase of the proportion of subpopulations contribute to the failure of CFD treatment in HR population.

**Table S1 The information of strains and plasmids used in this work.**

| Strains/plasmids | Genotype or characteristics | Source |
| --- | --- | --- |
| PAO1 | Wild-type strain, Gm^S^, Amp^R^ | Our lab |
| prop200 | Control plasmid, Gm^S^ | Our lab |
| prop200-vgrG | Overexpress plasmid, Gm^S^ | This work |

GmR and AmpR stand for gentamycin and ampicillin resistance, respectively.

**Table S2 Sequences of RNA and DNA Primers.**

| Name | Sense primer (5'-3') | Antisense primer (5'-3') | Source |
| --- | --- | --- | --- |
| *vgrG* cloning | atcggctcgtataatgaattcCAGTAGAAGGAACCGTCGA | atcggctcgtataatgaattcCAGTAGAAGGAACCGTCGA | This work |

**Table S3 The common mutations of gene in subpopulations detected by WGS.**

| Mutation | CRPA (rate) | CRE (rate) | CRAB (rate) | Total (rate) |
| --- | --- | --- | --- | --- |
| SNP | 14 (82.4%) | 11 (16.7%) | 17 (27.4%) | 42 (29.0%) |
| Insertion | 1 (5.9%) | 0 (0%) | 0 (0%) | 1 (0.7%) |
| Deletion | 2 (11.8%) | 46 (69.7%) | 39 (62.9%) | 87 (60.0%) |
| Duplication | 0 (0%) | 9 (13.6%) | 6 (9.7%) | 15 (10.3%) |
| Total | 17 (100%) | 66 (100%) | 62 (100%) | 145 (100%) |

CRPA, CRE, and CRAB stand for carbapenem-resistant *Pseudomonas aeruginosa*, carbapenem-resistant *Enterobacteriaceae,* carbapenem-resistant *Acinetobacter baumannii,* respectively.
